# The Role of Electronic Medical Record Systems and Pharmacovigilance Databases in the Surveillance of Antimicrobial Use and Resistance in Low- and Middle-Income Countries: Protocol for a Multistudy Project

**DOI:** 10.2196/81459

**Published:** 2026-04-28

**Authors:** Hager Saleh, Cecilia Stålsby Lundborg, Megha Sharma

**Affiliations:** 1 Department of Global Public Health, Karolinska Institutet, Tomtebodavägen 18 A, Stockholm, 17177, Sweden; 2 Department of Pharmacology, Ruxmaniben Deepchand Gardi Medical College, Surasa, Ujjain, 456006, India

**Keywords:** antimicrobial resistance, antibiotic resistance, electronic medical records, pharmacovigilance, digital health, antibiotic resistance surveillance, low- and middle-income countries, adverse drug reactions, VigiBase, antimicrobial resistance surveillance, AMR surveillance, digital intervention

## Abstract

**Background:**

Addressing antimicrobial resistance (AMR) requires proactive and innovative approaches in the digital world. The absence of comprehensive AMR data in low- and middle-income countries (LMICs) emphasizes the need for enhanced surveillance systems.

**Objective:**

This project aims to assess the implications of using digital tools, specifically electronic medical record (EMR) systems and pharmacovigilance (PV) databases (ie, VigiBase), as additional sources for the surveillance of antibiotic use and resistance patterns in LMICs.

**Methods:**

The project comprises 5 interconnected studies. Study 1 is a mixed methods study conducted in 3 private-sector tertiary hospitals in India. Data collection includes questionnaires and semistructured interviews with health care professionals and administrative staff. Quantitative data will be analyzed using descriptive statistics and qualitative data using reflexive thematic analysis. Study 2 is a retrospective cross-sectional study descriptively analyzing antibiotic-related individual case safety reports from LMICs submitted to VigiBase. Study 3 is a cross-sectional survey assessing health care professionals’ knowledge, attitudes, practices, and perceived barriers regarding antibiotic-related adverse drug reaction reporting in India. Studies 4 and 5 are systematic reviews examining (1) digital interventions for antimicrobial surveillance and appropriate prescribing, and (2) the use of PV databases for AMR surveillance. Searches will be conducted in PubMed, MEDLINE (Ovid), Embase, CINAHL, and Web of Science. Eligibility criteria include studies evaluating digital tools or PV databases in relation to antibiotic use or resistance. Two independent reviewers will perform screening and data extraction. Findings will be synthesized narratively and categorized by intervention type and reported outcomes.

**Results:**

The findings of study 1, phase 1, were published in May 2025. Phase 2 survey data collection reached 400 participants as of March 2026, and phase 3 included 36 interviews with health care professionals conducted in July and August 2024. For study 2, data were extracted from VigiBase in January 2025; analysis was completed and submitted for publication in December 2025. For study 4, article extraction was completed in December 2024, and title and abstract screening is ongoing as of December 2025. Full-text screening is scheduled to take place starting in May 2026. Data collection for study 3 is scheduled between October 2026 and May 2027. Study 5 is scheduled for 2026 and 2027. The project received funding in 2021 from the Research Council of Norway (project 325985) and the Swedish Research Council (project 2021-00889).

**Conclusions:**

Digital tools such as EMR systems and PV databases can play a crucial role in collecting more information about antibiotic use and AMR. This project emphasizes the importance of multidimensional solutions and explores the use of PV and EMRs in LMICs for effective AMR surveillance. Leveraging these digital systems offers an opportunity to enhance knowledge and strengthen efforts in the fight against AMR.

## Introduction

Antimicrobial resistance (AMR) is unquestionably a significant public health threat [1]. Without a global response, AMR is projected to be associated with 169 million cumulative deaths between 2025 and 2050, of which 39.1 million would be directly attributable to AMR. By 2050, annual deaths associated with AMR are expected to reach 8.22 million, with 1.91 million of those being directly caused by resistant infections [2].

AMR has no boundaries and, hence, affects all populations, with more serious impact in low- and middle-income countries (LMICs) [3]. LMICs are home to approximately 85% of the world’s population, with the BRICS countries (Brazil, Russia, India, China, and South Africa) accounting for approximately 40% [3]. LMICs face high mortality rates due to poor hygiene and sanitation and overcrowded areas where infections spread easily, compounded by limited access to high-quality health care and unregulated antibiotic use [2,3]. Addressing these challenges requires investment in health care system infrastructure, antibiotic stewardship, adequate antibiotic access, and improved diagnostic capacity to ensure appropriate antimicrobial use [2].

The anti-AMR initiatives led by the World Health Organization and improvements in AMR surveillance systems in several countries are not sufficient. Therefore, more data are needed to identify interventions that may slow down the development of resistance and determine the impact of AMR on human health [4]. Our published studies found that strategies must address problems at various societal and health care system levels and change the behaviors of numerous stakeholders to effectively combat AMR [5-7]. It is possible to develop a deeper understanding of the factors that contribute to antibiotic use as well as potential countermeasures to AMR by better understanding the dynamics of antibiotic prescribing, use, and dispensing from the viewpoints of different stakeholders (ie, prescribers, pharmacists, and consumers [5]).

In a digitalized world, innovative approaches are needed on a local and national level to empower the public and professionals to be proactive rather than reactive [8]. Digital tools such as electronic medical records (EMRs) and pharmacovigilance (PV) databases can play a crucial role in antibiotic prescription surveillance, optimizing antibiotic use, improving prescribing patterns, and monitoring resistance trends, all of which are otherwise labor-intensive and cumbersome [9-14]. Strengthening health care systems and ensuring equitable access to effective antimicrobials could significantly reduce AMR-related mortality, potentially saving 92.02 million lives [2].

Among these digital innovations, EMRs serve as a valuable tool in health care facilities, providing a structured platform for managing patient health data and supporting antimicrobial stewardship efforts [9]. An EMR is a patient’s comprehensive health data entered through a computer, tablet, or mobile device and saved electronically at a health care facility [15]. It contains a patient’s medical history, disease history, treatment history, current clinical conditions, and all other information required to provide personalized health care at the hospital [15]. It also includes a summary of the record of prescribed medicines, including patterns of antibiotic use [9].

EMR systems are often preloaded with information on the most appropriate antibiotics recommended for a specific indication [16,17]. This information is based on the prescribing guidelines to support the rational prescribing of antibiotics [16,17]. The EMR system also provides an opportunity to accurately document adverse drug reactions (ADRs; including from antimicrobials) and warn clinicians about inadvertent rechallenges when there is a preexisting reaction [16,17]. EMR systems’ support tools, such as e-prescribing and clinical decision support, assist physicians in providing rational antibiotic therapy, thus reducing the potential development of AMR [9].

While EMR systems enhance the appropriate use of antibiotics within health care facilities, PV databases provide valuable insights into broader trends of antimicrobial misuse and resistance at a population level [9-12]. As the world implements various steps to combat the threat posed by rising AMR, PV databases may become an important part of the larger multidisciplinary techniques used for the surveillance of AMR [11,12,18]. PV is “the science of activities related to the detection, assessment, understanding, and prevention of adverse effects or other potential drug-related problems” [19]; consequently, continuous review of PV data can reveal trends in suspected resistance and antimicrobial overuse [11].

PV databases are one-of-a-kind sources of information about potential medicine misuse (including antimicrobials) [11,12]. According to the authors of an Indian study, the occurrence of ADRs from antibiotics leads to poor adherence to treatment, which can trigger the development of resistance and treatment failure [20]. As a result, reports of ADRs caused by antibiotics can aid in developing policies and regulations for optimal antibiotic use and, thus, aid in the fight against AMR [10-12]. The scientific community continues to propose new AMR monitoring methods; in the same context, PV data could be a viable source of information for antimicrobial stewardship programs [21].

Moreover, all health care providers are principally required to register ADRs as part of their professional responsibilities. Therefore, it is critical to raise their awareness of PV. For this purpose, the assessment of health care professionals’ (HCPs) knowledge, attitudes, and reporting practices for ADRs will crucially assist in tailoring interventions to improve their attitudes, thereby improving reporting [22].

Given the potential critical role of EMRs and PV databases in monitoring AMR and guiding policy decisions, further research is needed to understand their effectiveness in LMICs, where health care infrastructure and surveillance mechanisms are often less developed.

Building on the identified gaps in digital antimicrobial surveillance in LMICs, this multistudy project aims to explore how EMR systems and PV databases may contribute to strengthening antimicrobial use monitoring and AMR surveillance. The overall objective of the project is to explore the potential implications of integrating digital health tools into antimicrobial surveillance strategies in LMIC contexts using India as a focused case study.

The specific objectives are as follows:

- Study 1—to assess HCPs’ experiences, preparedness, and perceptions regarding EMR system implementation and its potential impact on antimicrobial prescribing practices and ADR documentation in tertiary care hospitals
- Study 2—to investigate whether PV data from VigiBase can be used to identify patterns suggestive of antibiotic misuse or resistance-related events in LMICs
- Study 3—to evaluate HCPs’ knowledge, attitudes, practices, and perceived barriers related to antimicrobial-associated ADR reporting in India
- Study 4—to systematically review the use of EMR systems and other digital health interventions for rational antimicrobial use and resistance surveillance in LMICs
- Study 5—to systematically review the use of PV databases as additional data sources for monitoring AMR and antibiotic misuse in LMICs

## Methods

### Overview

**Figure 1.**
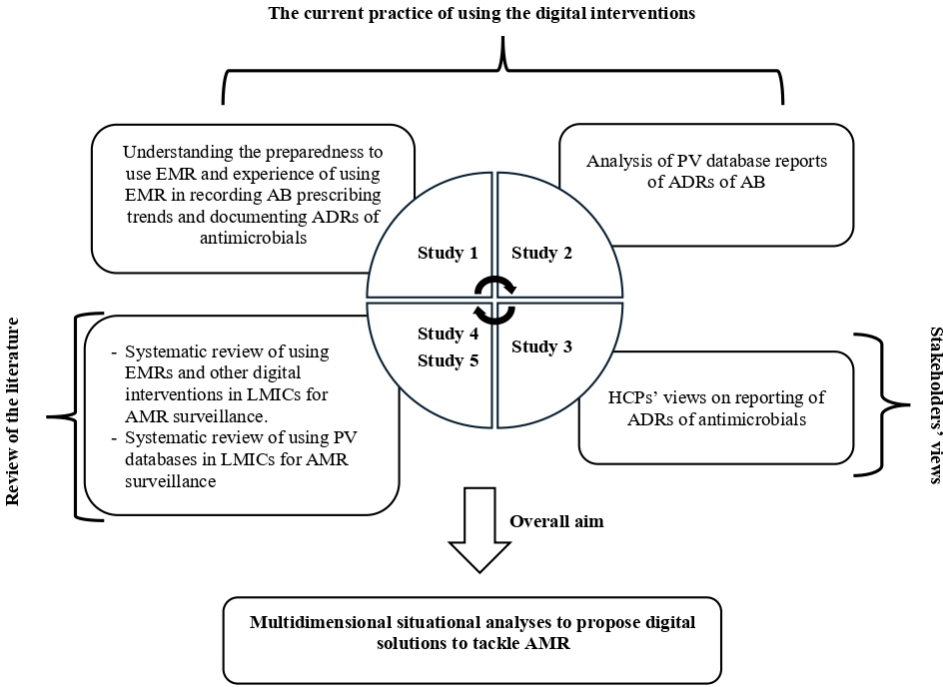
Overview of the 5 studies of the project. AB: antibiotic; ADRs: adverse drug reactions; AMR: antimicrobial resistance; EMR: electronic medical record; HCP: health care professional; LMICs: low- and middle-income countries; PV: pharmacovigilance.

This project is divided into 5 distinct but interrelated studies (Figure 1) designed to examine digital tools for antimicrobial surveillance in LMICs. For clarity and reproducibility, each study is described separately below.

### Study 1: EMR Implementation and Antimicrobial Use Surveillance

#### Study Design

Study 1 is a mixed methods study evaluating health care facilities’ preparedness and professionals’ experiences or perceptions regarding EMR implementation in India, with a focus on rational antibiotic use and ADR documentation. Data are collected through self-administered questionnaires and semistructured interviews. It consists of 3 phases:

- Phase 1—baseline assessment of EMR readiness and perceived barriers (published) [23], a cross-sectional study, and a self-administered questionnaire in 1 hospital that uses paper-based medical records
- Phase 2—expanded multifacility survey; building on phase 1, phase 2 expands data collection to 3 hospitals that use either paper-based medical records or partially implemented EMRs
- Phase 3—qualitative exploration to complement the survey findings; semistructured interviews were conducted

#### Study Settings

Three private-sector tertiary care hospitals located in the Ujjain district of Madhya Pradesh, India, were purposively selected for study 1. The facilities were chosen to enable comparison between institutions with differing levels of EMR implementation while maintaining comparability in ownership structure, service level (tertiary care), and patient population characteristics.

The hospitals were categorized as setting A (1 hospital with partially implemented EMR systems) and setting B (2 hospitals using paper-based medical records).

Although the facilities are located in the same district and share similar structural characteristics, they represent different stages of digital adoption within comparable institutional contexts. This design enables examination of how varying levels of EMR integration influence antimicrobial prescribing practices, ADR documentation, and preparedness for digital surveillance. While the study is geographically focused on India, the comparison between partial EMR implementation and paper-based records reflects common transitional scenarios observed in several health care settings in LMICs.

#### Study Participants and Data Collection Tools

The questionnaires are being distributed among HCPs and administrative staff working in the participating hospitals, including physicians, nurses, and relevant management personnel in both EMR and non-EMR settings. The questionnaires were developed through a structured literature review and expert consultation followed by pretesting for clarity and contextual relevance. Test-retest reliability was assessed, demonstrating substantial agreement (Cohen κ=0.85‐0.90).

The full instrument, including item structure and domains, was included as supplementary material in the phase 1 manuscript published in May 2025 [23], which also provides detailed information on sampling, piloting procedures, and survey validation [23].

Two structured survey instruments were developed for phase 2 tailored to the medical record status of settings A and B. The development of the expanded surveys was informed by findings from phase 1 and aligned with the study objectives, focusing on antimicrobial use, AMR surveillance, and ADR documentation.

#### Survey Structure and Content

Both structured surveys include domains as provided in Textbox 1.

The surveys consist primarily of multiple-choice, Likert-scale, and structured response items, with open-ended questions to capture contextual perspectives. Prior to implementation, the expanded instruments were reviewed by the research team and piloted with HCPs to assess clarity. Minor revisions were made before final deployment.

Textbox 1. **Overview of surveys structure and content.**

- Demographic and professional characteristics
- Institutional and digital infrastructure—availability of computers and internet access, type of medical record system in use, IT support availability, and data integration
- Antimicrobial use and prescription monitoring—availability and use of antimicrobial prescribing guidelines, monitoring and audit of antimicrobial prescriptions, the role of microbiology testing in prescribing decisions, and tracking of antibiotic consumption
- Antimicrobial resistance surveillance—monitoring resistance trends and documentation of resistance data
- Adverse drug reaction (ADR) reporting—current ADR documentation practices and the role of electronic medical record (EMR) systems in reporting
- EMR functionality (setting A only)—use of electronic prescribing and clinical decision support tools, alerts for drug interactions and allergies, integration with laboratory results and ability to track antimicrobial use and resistance data
- Preparedness and perceived barriers (setting B only)—organizational readiness for EMR implementation, technical and financial barriers, legal and regulatory concerns, and perceived benefits of adopting EMR systems

#### Semistructured Interviews

Semistructured interviews were conducted in July and August 2024 with a purposive subset of survey respondents, including physicians, nurses, and pharmacists from both setting A and setting B.

Separate interview guides were developed for each professional category (physicians, nurses, and pharmacists) in each setting (setting A and setting B), resulting in 6 interview guides tailored to record professional roles and digital context but following a comparable structure to allow for thematic comparison across groups. The interview guides were piloted and refined prior to full-scale data collection.

Interview domains included antibiotic prescribing decision-making processes; the role of microbiology testing in treatment decisions; current systems for antibiotic prescription monitoring; ADR documentation and reporting practices; antimicrobial stewardship activities; use of EMR functions (setting A), including electronic prescribing and decision support tools; and perceived barriers to and facilitators of EMR implementation (setting B).

#### Data Analysis

The survey data will be analyzed using descriptive and inferential statistical methods. Categorical variables will be summarized using frequencies and percentages, whereas continuous variables will be presented as means with SDs. Comparative analyses will be conducted to examine differences between setting A and setting B facilities, as well as across professional groups (eg, physicians, nurses, and pharmacists). For categorical variables, chi-square tests or Fisher exact tests, where appropriate, will be applied. For continuous variables, independent 2-tailed *t* tests or Mann-Whitney *U* tests will be used for 2-group comparisons, and one-way ANOVA or Kruskal-Wallis tests will be used for comparisons involving more than 2 groups, as appropriate.

Where relevant, multivariable analyses may be performed to explore associations among institutional digital status; professional characteristics; and key outcomes such as EMR preparedness, antibiotic prescribing monitoring practices, and AMR surveillance activities. A *P* value of less than .05 will be considered statistically significant. All analyses will be conducted at the aggregated group level to ensure respondent anonymity.

The qualitative data are being analyzed using reflexive thematic analysis [24]. Coding is being conducted iteratively, with regular discussions among the research team to refine interpretations and ensure analytic rigor. To enhance trustworthiness, several strategies will be applied. Credibility will be strengthened through iterative coding and team discussions. Dependability will be enhanced through independent coding by 2 researchers, followed by comparison and discussion of coding decisions to reach analytic consensus. Discrepancies will be discussed within the research team to refine theme development and ensure transparency in the analytic process. Transferability will be supported by providing detailed descriptions of the study context and participant characteristics.

#### Ethical Considerations

Study 1 is part of the multicentric project “EquityAMR – Digital equity in AMR policy and practice – Building equity in digital global health: the case of antimicrobial resistance in LMICs,” and ethics approval was granted by the ethics committee of RD Gardi Medical College, Ujjain (reference 17/2022).

Written and oral informed consent was obtained. Confidentiality is strictly maintained throughout data storage and analysis. All identifiable variables will be either anonymized or removed prior to analysis. Access to both digital and physical data will be restricted to authorized personnel on a need-to-know basis to minimize unnecessary exposure. Data will be stored in India on password-protected devices with authenticated access.

The audio recordings and original transcripts are securely stored by the principal investigator (MS). Anonymized versions of the transcripts are being analyzed by authorized researchers. Any summaries or direct quotations used in reporting will be anonymized to ensure that participants cannot be identified. No compensation was provided to any of the participants.

### Study 2: PV Databases as Additional Tools for AMR Surveillance

#### Study Design

Study 2 is a retrospective cross-sectional study analyzing individual case safety reports (ICSRs) related to antibiotics submitted to the global PV database (ie, VigiBase).

#### Data Source

The study used ICSRs related to systemic antibiotics submitted from LMICs via VigiBase to the World Health Organization’s global PV database maintained by the Uppsala Monitoring Centre [21]. Antibiotic-related ICSRs were identified using Anatomical Therapeutic Chemical classification codes, and relevant ADRs were retrieved using predefined Medical Dictionary for Regulatory Activities Preferred Terms associated with antibiotic resistance, treatment failure, and potential misuse.

#### Data Analysis

A descriptive quantitative analysis was conducted to characterize reporting patterns. Variables analyzed included year of reporting, reporter type, suspected antibiotic, and reported Medical Dictionary for Regulatory Activities Preferred Terms. The analysis aimed to describe the reported resistance-related events and potential indicators of inappropriate antibiotic use within PV data from LMICs. Data analysis has been completed, and the manuscript was submitted for publication in December 2025.

#### Ethical Considerations

Study 2 did not require ethics approval as the data received from the VigiBase database were completely anonymized and had no individual identifiers before extraction.

### Study 3: Knowledge, Attitudes, and Practices Regarding ADR Reporting

#### Study Design

A cross-sectional study design will be used to assess the knowledge, attitudes, practices, and perceived barriers among HCPs in India concerning the reporting of ADRs related to antimicrobial use.

#### Participants and Data Collection Tool

HCPs working in the same tertiary care hospitals as in study 1 will be eligible. A self-administered questionnaire was developed through literature review and expert consultation to ensure content validity. The questionnaire will be distributed among HCPs, including physicians, nurses, and pharmacists.

The questionnaire consists of multiple sections. It covers participants’ demographic information; the knowledge domain includes multiple-choice questions assessing understanding of PV concepts, ADR definitions, reporting requirements under the Pharmacovigilance Programme of India, antibiotic misuse, AMR, and signal detection. The attitudes and practices sections include closed-ended (“yes,” “no,” or “don’t know”) statements evaluating perceptions of ADR reporting responsibilities, previous reporting behavior, training exposure, and perceived importance of PV activities. The barrier section includes multiple-response items exploring factors that may discourage ADR reporting. In addition, open-ended questions are included to gather suggestions for interventions to improve ADR reporting practices.

#### Data Analysis

Survey data will be analyzed using descriptive and inferential statistical methods. Categorical variables will be summarized using frequencies and percentages, whereas continuous variables will be presented as means with SDs or medians with IQRs as appropriate. Composite scores may be calculated for knowledge, attitudes, and practices domains where applicable. Comparisons across professional groups (eg, physicians, nurses, and pharmacists) will be performed using chi-square tests for categorical variables and one-way ANOVA. Multivariable analyses may be conducted to identify factors associated with ADR reporting practices and perceived barriers. Responses to open-ended questions will be reviewed and categorized thematically to complement the quantitative findings.

#### Ethical Considerations

Study 3 falls under the same ethics approval as study 1 (reference 17/2022). Informed consent procedures and confidentiality measures similar to those for study 1 will be followed.

### Study 4: Systematic Review of Digital Interventions for AMR Surveillance in LMICs

#### Study Design

This study will be a systematic literature review to identify any digital interventions used in LMICs for AMR surveillance and appropriate antimicrobial prescribing.

#### Information Sources and Search Strategy

Electronic databases searched included PubMed, MEDLINE (Ovid), Embase (Elsevier), CINAHL, and Web of Science. Key search terms included “antibiotics,” “antibiotic resistance,” “electronic medical records,” “antibiotics use,” “antibiotics prescription,” and “digital technology interventions.” The review is being conducted and will be reported in accordance with the PRISMA (Preferred Reporting Items for Systematic Reviews and Meta-Analyses) guidelines. The detailed search strategy and exclusion and inclusion criteria are available in the protocol, which has been registered at PROSPERO (CRD42024578136).

#### Study Selection and Data Extraction

Four reviewers are independently screening titles and abstracts, followed by full-text review.

Data extraction will be conducted using a standardized data extraction form that will be developed for this review based on predefined variables. Extracted variables will include study characteristics (author, year, country, and study design), setting and population, type of digital intervention, outcomes of the interventions, and reported implementation challenges or facilitators. The extracted data will be cross-checked between reviewers to ensure accuracy and completeness.

#### Risk of Bias

Discrepancies between reviewers will be resolved through discussion or consultation with an additional reviewer. The quality of the included studies will be assessed using the Newcastle-Ottawa Scale for observational studies and the Cochrane risk-of-bias tool for randomized trials. Assessment of meta-bias, such as publication bias, will be considered qualitatively by examining study characteristics, reporting completeness, and potential selective outcome reporting. Due to the expected heterogeneity in study designs, interventions, and reported outcomes, a quantitative meta-analysis is not anticipated. The overall strength and certainty of the body of evidence will be assessed using the Grading of Recommendations Assessment, Development, and Evaluation approach.

#### Data Analysis

Findings from identified studies will be synthesized and categorized by type of digital intervention, reported outcomes, and implementation context to provide an overview of digital tools used by LMICs for antimicrobial surveillance and appropriate use of antimicrobials.

#### Ethical Considerations

Ethics approval is not required as this study involves a systematic review of published literature. The review protocol was registered in August 2024 at PROSPERO (CRD42024578136).

### Study 5: Systematic Review of Using PV Databases for AMR Surveillance

#### Study Design

This study is a systematic literature review to assess the use of PV databases for AMR surveillance and monitoring of antibiotic misuse, including resistance-related ADRs.

#### Information Sources and Search Strategy

Electronic databases to be searched include PubMed, MEDLINE (Ovid), Embase (Elsevier), CINAHL, and Web of Science. Key search terms include “antibiotics,” “adverse drug reactions,” “pharmacovigilance,” “antibiotic resistance,” “antibiotic misuse,” and “off-label use.” The review will be conducted and reported in accordance with PRISMA guidelines. The full search strategy and exclusion and inclusion criteria will be documented in the review protocol, which will be registered in PROSPERO.

#### Study Selection and Data Extraction

Two reviewers will independently screen titles and abstracts, followed by full-text assessment. Data extraction will be conducted using a standardized extraction form. Extracted variables will include study characteristics (author, year, country, and study design), type of PV database used (eg, national or international), study population, antibiotics investigated, reported resistance- or misuse-related terms, analytical methods applied, and key findings. Extracted data will be cross-checked between reviewers to ensure accuracy and completeness.

#### Risk of Bias

Discrepancies between reviewers will be resolved through discussion or consultation with a third reviewer. The quality of the included studies will be assessed using the Newcastle-Ottawa Scale for observational studies and the Cochrane risk-of-bias tool for randomized trials. Potential meta-biases, including publication bias and selective reporting, will be considered qualitatively. Due to the expected heterogeneity in study designs and reported outcomes, a quantitative meta-analysis is not anticipated. The overall strength and certainty of the body of evidence will be assessed using the Grading of Recommendations Assessment, Development, and Evaluation approach.

#### Data Analysis

Findings from the included studies will be synthesized narratively and categorized according to the type of PV database used, resistance-related terms identified, methodological approaches applied, and implementation context within LMIC settings. Where feasible, patterns in database use and reported surveillance outcomes will be summarized to provide an overview of the role of PV systems in AMR monitoring.

#### Ethical Considerations

Ethics approval is not required as this study involves secondary analysis of published literature. The review protocol will be registered in PROSPERO.

### Dissemination

The findings from these studies will be disseminated through academic publications, presentations at international conferences, and stakeholder engagement activities to inform policymakers for the development of AMR surveillance and best practices in AMR surveillance using digital tools in LMICs.

## Results

### Overview

The preproject phase lasted 1 year and involved project and study planning, as well as obtaining ethics approval. Funding was obtained in 2021 from the Research Council of Norway and the Swedish Research Council. The project spans 5 years, covering the planning, development of data collection tools, data collection and analysis, and dissemination of findings for all the studies (Table 1).

**Table 1.** Project timeline (2022‐2027)^a^.

Study and phase	2022	2023	2024	2025	2026	2027
Preproject phase	🟩					
Study 1						
Phase 1		🟩	🟩	🟩		
Phase 2			🟨	🟨	🟨	
Phase 3			🟨	🟨	🟨	
Study 2		🟩	🟩	🟩		
Study 3					🟥	🟥
Study 4			🟨	🟨	🟨	
Study 5					🟥	🟥

a Color code: green=completed, yellow=study started and ongoing, and red=study not started yet.

### Study 1

The survey for phase 1 was developed and pilot-tested in 2023, and the findings have been published [23]. In phase 1 (n=105 medical doctors), 92.4% (97/105) of respondents supported the implementation of EMRSs. Major perceived barriers included lack of IT personnel (59/101, 58.4%), staff shortages (57/102, 55.9%), staff resistance (51/99, 51.5%), insufficient infrastructure (47/101, 46.5%), and financial constraints (43/99, 43.4%) [23]. Technological challenges such as unreliable internet access (42/100, 42%) were also highlighted. At the same time, respondents anticipated key benefits, including improved accessibility of clinical data (77/98, 78.6%), enhanced operational efficiency (78/99, 79%), improved quality monitoring (81/101, 80.2%), and strengthened treatment planning (77/103, 74.8%) [23].

Building on these findings, phase 2 involved an expanded survey across 3 hospitals (settings A and B). The survey was developed and pilot-tested in 2023, and data were collected until March 2026; 400 participants were enrolled. Data analysis has not started yet.

For phase 3, interview guides were developed and pilot-tested between March 2024 and June 2024. Semistructured interviews were conducted with 36 HCPs in July 2024 to August 2024 and audio recorded following informed consent. Recordings were transcribed verbatim, and anonymized transcripts will be used for qualitative analysis.

### Study 2

Data were requested from the Uppsala Monitoring Centre. The dataset was extracted and received in January 2025. The analysis has since been completed, and the findings were submitted for publication in December 2025.

### Study 3

The first draft of the questionnaire was developed in 2026, and it will be pilot-tested in May 2026. The survey is scheduled to be conducted between October 2026 and May 2027.

### Study 4 and Study 5

Keywords for both systematic reviews were identified in August 2024. Article extraction was completed in December 2024 for study 4, and title and abstract screening continued up to December 2025. Full-text screening is scheduled to take place starting in May 2026.

Study 5 has not started yet. It is planned to commence in 2026 to 2027.

## Discussion

### Expected Findings

Recognizing the growing burden of AMR and the urgent need for strengthened surveillance infrastructures in resource-limited settings, this project adopts a comprehensive and multimethod approach organized into 5 interlinked studies. Together, these studies aim to address critical gaps in antibiotic use monitoring, resistance trend detection, and reporting of ADRs from antimicrobials using digital tools as central enablers. Specifically, the project investigates the potential implications of EMR systems and PV databases in enhancing the surveillance of antimicrobial use and AMR patterns in LMICs, with a focus on India as a representative case. Each study is designed to address critical gaps, challenges, and opportunities associated with AMR surveillance. These include limited data on prescribing practices, underreporting of ADRs, and lack of integration between digital health tools and existing surveillance platforms. By integrating digital interventions with established AMR surveillance practices, this research aims not only to explore the current capacities and limitations of EMR and PV systems but also to identify strategies for optimizing their use across diverse health care settings.

This work contributes to growing global efforts to move toward more data-driven AMR containment strategies, recognizing the need to strengthen health systems’ ability to detect resistance patterns early, track inappropriate antimicrobial use, and promote rational prescribing practices. Eventually, the project aspires to generate actionable insights that can inform better management of antimicrobial prescriptions; enable more accurate and timely monitoring of resistance trends; and support the development of more robust, responsive, and sustainable public health systems in LMICs.

### Public Health Significance

AMR poses a significant public health challenge, particularly in LMICs, where the prevalence of infectious diseases is high and health care infrastructure is often underresourced. The inappropriate use of antimicrobials, alongside inadequate sanitation and limited infection control measures, creates conditions encouraging the proliferation of antimicrobial-resistant microorganisms [25]. Consequently, populations in these regions face an elevated risk of drug-resistant infections, resulting in increased morbidity and mortality rates and significant economic burden [25].

The integration of digital tools such as EMR systems and reporting to PV databases in health care settings offers a promising approach to addressing these challenges. EMR systems allow for real-time documentation of antimicrobial prescriptions and treatment pathways, offering health care providers access to decision support information that may reduce inappropriate use [26]. Additionally, EMRs facilitate the accurate documentation of ADRs and therapeutic failure, both of which are crucial for ensuring patient safety and tracking antibiotic effectiveness [26].

PV databases, on the other hand, can complement EMR data by providing broader postmarketing surveillance insights. They offer valuable resources for monitoring trends in antibiotic resistance and misuse [21]. Collecting and systematically analyzing these data can help identify emerging resistance patterns, contributing to more informed public health decisions and policymaking and guiding national responses.

The proposed project will provide a crucial lens through which the challenges and opportunities of digital interventions in LMICs can be examined. India is the most populated country in the world, with high antibiotic consumption and significant diversity in health care delivery, ranging from well-equipped urban hospitals to underresourced rural clinics. Therefore, the project aims to study India’s diverse health care settings, which offer a microcosm of LMIC challenges and opportunities in adopting strategies and tools to control AMR [27].

### New Knowledge Generation

This project will generate new insights into the use of digital tools such as EMR systems and PV databases in combating AMR. Specifically, it will (1) assess the experiences and readiness of health care facilities in India regarding EMR implementation, offering practical recommendations for future deployments; (2) investigate the potential of PV databases to track antibiotic resistance trends and inappropriate antibiotic use, thereby highlighting their role in antimicrobial stewardship programs; (3) evaluate HCPs’ knowledge, attitudes, and practices related to ADR reporting, identifying barriers to and facilitators of effective PV practice; (4) provide a comprehensive overview of the present status of digital tools in LMICs, identifying gaps and opportunities for improvement, and highlight recent national and institutional policy changes aimed at addressing AMR, including efforts to integrate digital surveillance tools into health systems; and (5) identify global initiatives aimed at strengthening PV systems and examine how LMICs can adopt these approaches to improve ADR reporting and antibiotic use monitoring, thereby supporting AMR containment efforts.

Together, these outputs aim to bridge current knowledge gaps and guide future efforts in digital transformation, capacity building, and policy development to strengthen AMR surveillance infrastructures in LMICs.

### Conclusions

This project underscores the critical role of digital tools in enhancing AMR surveillance in LMICs. Examining the potential of EMR systems and PV databases will provide actionable insights to optimize antibiotic use and improve patient safety. The findings are expected to inform policy recommendations and practical interventions, eventually contributing to a more robust and responsive AMR surveillance infrastructure. The emphasis on a multidimensional approach integrating technology with health care practice highlights the need for collaborative efforts in tackling the global challenge of AMR.

## Supplementary material

10.2196/81459 Checklist 1 PRISMA-P checklist.

## Abbreviations

ADR: adverse drug reaction
AMR: antimicrobial resistance
EMR: electronic medical record
HCP: health care professional
ICSR: individual case safety report
LMIC: low- and middle-income country
PRISMA: Preferred Reporting Items for Systematic Reviews and Meta-Analyses
PRISMA-P: Preferred Reporting Items for Systematic Reviews and Meta-Analyses Protocols
PV: pharmacovigilance

## References

[1] de Kraker ME, Stewardson AJ, Harbarth S (2016). Will 10 million people die a year due to antimicrobial resistance by 2050?. PLoS Med.

[2] GBD 2021 Antimicrobial Resistance Collaborators (2024). Global burden of bacterial antimicrobial resistance 1990-2021: a systematic analysis with forecasts to 2050. Lancet.

[3] Sulis G, Sayood S, Gandra S (2022). Antimicrobial resistance in low- and middle-income countries: current status and future directions. Expert Rev Anti Infect Ther.

[4] EClinicalMedicine (2021). Antimicrobial resistance: a top ten global public health threat. EClinicalMedicine.

[5] Saleh HA, Borg MA, Stålsby Lundborg C, Saliba-Gustafsson EA (2022). General practitioners’, pharmacists’ and parents’ views on antibiotic use and resistance in Malta: an exploratory qualitative study. Antibiotics (Basel).

[6] Sharma M, Joshi R, Shah H, Macaden R, Lundborg CS (2015). A step-wise approach towards introduction of an alcohol based hand rub, and implementation of front line ownership- using a, rural, tertiary care hospital in central India as a model. BMC Health Serv Res.

[7] Diwan V, Gustafsson C, Rosales Klintz S (2016). Understanding healthcare workers self-reported practices, knowledge and attitude about hand hygiene in a medical setting in rural India. PLoS One.

[8] Beerlage-de Jong N, van Gemert-Pijnen L, Wentzel J, Hendrix R, Siemons L (2017). Technology to support integrated antimicrobial stewardship programs: a user centered and stakeholder driven development approach. Infect Dis Rep.

[9] Kamaluddin R, Adisasmita WB The role of electronic medical record in enhancing rational antibiotics prescription: a systematic review.

[10] Habarugira JM, Figueras A (2021). Antimicrobial stewardship: can we add pharmacovigilance networks to the toolbox?. Eur J Clin Pharmacol.

[11] Habarugira JM, Härmark L, Figueras A (2021). Pharmacovigilance data as a trigger to identify antimicrobial resistance and inappropriate use of antibiotics: a study using reports from The Netherlands Pharmacovigilance Centre. Antibiotics (Basel).

[12] Habarugira JM, Figueras A (2021). Pharmacovigilance network as an additional tool for the surveillance of antimicrobial resistance. Pharmacoepidemiol Drug Saf.

[13] Machowska A, Sparrentoft J, Dhakaita SK, StålsbyLundborg C, Sharma M (2019). Perioperative antibiotic prescribing in surgery departments of two private sector hospitals in Madhya Pradesh, India. Perioper Med (Lond).

[14] Skender K, Singh V, Stalsby-Lundborg C, Sharma M (2021). Trends and patterns of antibiotic prescribing at orthopedic inpatient departments of two private-sector hospitals in Central India: a 10-year observational study. PLoS One.

[15] Aldosari B (2017). Patients’ safety in the era of EMR/EHR automation. Inform Med Unlocked.

[16] McLachlan G, Broomfield A, Elliott R (2023). Completeness and accuracy of adverse drug reaction documentation in electronic medical records at a tertiary care hospital in Australia. Health Inf Manag.

[17] Jung IY, Kim JJ, Lee SJ (2017). Antibiotic-related adverse drug reactions at a tertiary care hospital in South Korea. Biomed Res Int.

[18] Habarugira JM, Härmark L, Figueras A (2021). Adverse drug reaction reports containing AMR-relevant MedDRA terms in the Dutch Pharmacovigilance database. Preprints.org.

[19] Pharmacovigilance: overview. European Medicines Agency.

[20] Sharma M, Baghel R, Thakur S, Adwal S (2021). Surveillance of adverse drug reactions at an adverse drug reaction monitoring centre in Central India: a 7-year surveillance study. BMJ Open.

[21] (2017). Antimicrobial resistance-an overlooked adverse event. https://who-umc.org/media/2775/web_uppsalareports_issue74.pdf.

[22] Saleh HA, Figueras A, Fourrier-Réglat A (2016). Knowledge, attitude and practice of health professionals towards adverse drug reactions reporting. Eur J Pharm Med Res.

[23] Saleh H, Lundborg CS, Sharma M (2025). Perceived benefits and barriers of medical doctors regarding electronic medical record systems in an Indian private-sector healthcare facility. BMC Health Serv Res.

[24] Braun V, Clarke V (2023). Toward good practice in thematic analysis: avoiding common problems and be(com)ing a *knowing* researcher. Int J Transgend Health.

[25] Sultana M, Perves N, Uddin N, Chowdhury ME, Amin N (2024). The vicious impact of antimicrobial resistance on global public health security and the role of healthcare systems and policy in combating AMR. World J Public Health.

[26] van den Broek AK, Beishuizen BH, Haak EA (2021). A mandatory indication-registration tool in hospital electronic medical records enabling systematic evaluation and benchmarking of the quality of antimicrobial use: a feasibility study. Antimicrob Resist Infect Control.

[27] Mukherjee A (2021). Implementing electronic health records in India: status, issues & way forward. Biomed J Sci Technol Res.

[28] (2023). 22nd ISoP Annual Meeting “Putting Patients First in Pharmacovigilance: International Perspectives from Global South” 6–9 November 2023 Bali, Indonesia. Drug Saf.

